# Comparison of sodium ion levels between an arterial blood gas analyzer and an autoanalyzer in preterm infants admitted to the neonatal intensive care unit: a retrospective study

**DOI:** 10.1186/s12887-016-0636-4

**Published:** 2016-07-20

**Authors:** Hyunho Kim, Jin Kyu Kim, Soo Chul Cho

**Affiliations:** Department of Pediatrics, Chonbuk National University Hospital, 20, Geonji-ro, Deokjin-gu, Jeonju, 54907 South Korea; Research Institute of Clinical Medicine of Chonbuk National University-Biomedical Research Institute of Chonbuk National University Hospital, Jeonju, South Korea

**Keywords:** Blood gas analysis, Hyponatremia, Preterm infant, Protein, Sodium

## Abstract

**Background:**

The difference in sodium ion levels determined with direct and indirect methods often exceeds the permissible limit clinically. Additionally, no previous study has assessed the difference in the sodium ion levels between direct and indirect methods in premature infants. Therefore, the present study aimed to compare sodium ion levels obtained using an arterial blood gas analyzer (ABGA; direct method) and an autoanalyzer (indirect method) to determine whether they are equivalent in premature infants.

**Methods:**

The present retrospective study included 450 preterm infants (weight, <2500 g) who were admitted to the neonatal intensive care unit (NICU) of our hospital between March 2012 and April 2014. We compared sodium ion levels in 1041 samples analyzed using an ABGA (Stat Profile® CCX Series, Nova Biomedical, Waltham, MA) and an autoanalyzer (ADVIA® 2400 Clinical Chemistry System, Siemens, Tarrytown, NY). The data were evaluated using Spearman’s correlation coefficient analysis, Bland-Altman plot, Deming regression analysis, and multivariate logistic regression analysis.

**Results:**

The mean sodium ion levels were 134.6 ± 3.5 mmol/L using the ABGA and 138.8 ± 4.7 mmol/L using the autoanalyzer (*P* < 0.001). Among the 1041 samples, 957 (91.9 %) showed lower sodium ion levels with the ABGA than with the autoanalyzer and 74 (7.1 %) showed lower sodium ion levels with the autoanalyzer than with the ABGA. The incidence of hyponatremia identified using the ABGA was 51.9 % (541/1041), while the incidence of hyponatremia identified using the autoanalyzer was only 14.0 % (146/1041). The Deming regression analysis of the sodium ion levels between the ABGA and the autoanalyzer yielded the following formula: autoanalyzer Na (mmol/L) = 20.7 + (0.9 × ABGA Na [mmol/L]). In the multivariate logistic regression analysis, low plasma protein level (<4.3 g/dL) was found to be an independent risk factor for a sodium ion level difference of >4 mmol/L between the two methods (odds ratio = 2.870, *P* < 0.001).

**Conclusion:**

The sodium ion levels determined using the ABGA and the autoanalyzer might not be equivalent in premature infants admitted to the NICU. Therefore, clinicians should be careful when diagnosing sodium ion imbalance in premature infants and providing treatment.

**Electronic supplementary material:**

The online version of this article (doi:10.1186/s12887-016-0636-4) contains supplementary material, which is available to authorized users.

## Background

Sodium is an essential electrolyte in the intracellular and extracellular fluids of the body. Metabolism is closely associated with sodium, and an imbalance in the sodium ion levels can result in serious life-threatening conditions [1–3]. In the neonatal intensive care unit (NICU), preterm infants are at high risk for an imbalance in the sodium ion levels because of renal immaturity, renal failure, diuretics use, and fluid management. Therefore, it is very important to frequently determine sodium ion levels in preterm infants.

Direct and indirect ion-sensitive electrodes have been used for electrolyte analysis in recent years. The direct electrode analyzes undiluted whole blood. An arterial blood gas analyzer (ABGA) incorporates a direct electrode. An ABGA is useful method to evaluate baby constantly and requires small amounts of blood. The indirect electrode, incorporated in an automated analyzer, analyzes diluted plasma. It is generally used in central hospital laboratories.

The United States Clinical Laboratory Improvement Amendments (US CLIA) 1988 rules allow for a difference of up to 4 mmol/L in the sodium ion levels [4]. However, the difference in the sodium ion levels between the two types of methods often exceeds the limit clinically. The difference in the sodium ions levels could be caused by a variety of factors, including equipment, transport containers, albumin levels, protein levels, and the patient’s medical condition [5–8]. No previous study has assessed the difference in the sodium ion levels between the direct and indirect methods in premature infants. We hypothesized that sodium ion levels obtained using an autoanalyzer (indirect method) and an ABGA (direct method) are equivalent. The present study aimed to compare sodium ion levels between an autoanalyzer and an ABGA to test this hypothesis.

## Methods

The present study included inborn preterm infants (weight, <2500 g) who were admitted to the NICU of Chonbuk National University Hospital between March 2012 and April 2014. We retrospectively analyzed the patient data from the database of the NICU and identified 450 patients who underwent arterial blood collection for electrolyte analysis using both an ABGA and an autoanalyzer on admission and every week after the hospitalization to evaluate sodium level differences at different time points of sample collection.

Arterial blood samples were collected simultaneously for measurements with an ABGA and an autoanalyzer. One sample was collected in a dry heparin syringe (BD Preset^TM^, BD Diagnostics, Plymouth, UK) and was analyzed using an ABGA (Stat Profile® CCX Series, Nova Biomedical, Waltham, MA). The ABGA undergoes automatic two-point calibration every 2, 4, or 6 h and single-point calibration every 30 min or after each sample. Another sample was collected in a microtube (Microtainer^TM^ tubes #365978, BD Diagnostics) and was analyzed using a central autoanalyzer (ADVIA® 2400 Clinical Chemistry System, Siemens, Tarrytown, NY). The autoanalyzer also has an autocalibration system, which checks the accuracy and precision using a sample buffer for sodium. If the measurement is outside the expected range, the autocalibration system posts an alarm message, and the solution is re-analyzed. The samples were immediately sent to our central laboratory and were analyzed. The autoanalyzer simultaneously reports plasma constituents, including plasma protein and albumin. A total of 1041 samples were analyzed using the ABGA and the autoanalyzer.

The data were interpreted based on the indirect sodium level. A serum sodium ion level of 135–145 mmol/L was considered normal. Patients with a serum sodium ion level <135 mmol/L were diagnosed with hyponatremia, and those with a serum sodium ion level >145 mmol/L were diagnosed with hypernatremia.

### Statistical methods

Means, standard deviations, and coefficients of variation were calculated. A paired *t*-test was used to compare the sodium ion levels of the ABGA and the autoanalyzer. A Bland-Altman analysis was performed to assess the differences in the sodium ion levels between the ABGA and the autoanalyzer. We defined a sodium ion level of 4 mmol/L as the acceptable upper limit for the difference between the two methods [4]. Deming regression analysis and a Bland-Altman plot were used to evaluate the sodium ion levels between the ABGA and the autoanalyzer. Logistic regression analysis was performed to identify the risk factors for a high difference in the sodium ion levels between the ABGA and the autoanalyzer. The factors assessed were gestational age, birth body weight, protein levels, and albumin levels. All statistical analyses were performed using SPSS version 18.0 (IBM Corp., Armonk, NY) and MedCalc Statistical Software version 15.8 (MedCalc Software, Mariakerke, Belgium). A *P*-value <0.05 was considered to indicate a statistically significant difference.

## Results

**Fig. 1:**
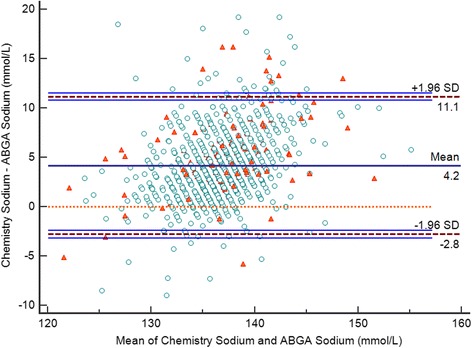
Bland-Altman plot of sodium ion levels determined using the arterial blood gas analyzer and autoanalyzer. The horizontal axis shows the mean of the sodium ion level determined using the blood chemistry test and the sodium ion level measured with the ABGA, while the differences in the sodium ion levels between the arterial blood gas analyzer and the autoanalyzer are presented on the Y-axis. Circles indicate low serum protein level (<4.3 g/dL), while triangles indicate high serum protein level (≥4.3 g/dL)

**Fig. 2:**
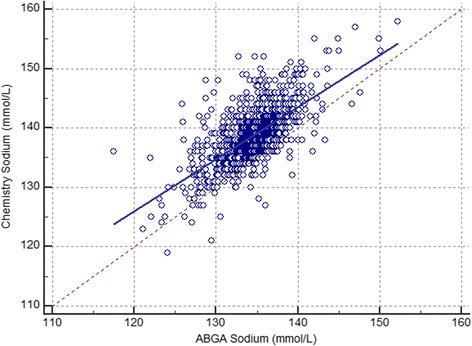
Deming fit (solid blue line) for sodium ion levels. The sodium ion levels determined using the arterial blood gas analyzer (ABGA) are presented on the X-axis, while the sodium ion levels determined using the chemistry are presented on the Y-axis. Deming regression equation: chemistry Na (mmol/L) = 20.7 + (0.9 × ABGA Na [mmol/L]), *R* ^2^ = 0.43

The study analyzed data from 450 infants (1041 samples) admitted to the NICU. The gestational age of the infants was 31.4 ± 3.5 weeks, and the body weight of the infants was 1605.4 ± 513.3 g. The average time of sample collection was 10.0 ± 12.9 days after the admission to the NICU. Samples were collected on admission and every week. The sodium ion levels did not differ between time points of sample collection. The sodium ion levels were 134.6 ± 3.5 mmol/L using the ABGA and 138.8 ± 4.7 mmol/L using the autoanalyzer (Table 1). The mean difference was 4.2 ± 3.6 mmol/L (*P* < 0.001), correlation coefficient (R) was 0.662, and adjusted R^2^ was 0.43 with a 95 % confidence interval of 3.96–4.39. Among the 1041 samples, 957 (91.9 %) showed lower sodium ion levels with the ABGA than with the autoanalyzer and 74 (7.1 %) showed lower sodium ion levels with the autoanalyzer than with the ABGA. A Bland-Altman comparison of sodium ion levels between the ABGA and the autoanalyzer showed that the limits of agreement were −2.8 to 11.1 (Fig. 1). The Deming regression analysis of the sodium ion levels between the ABGA and the autoanalyzer yielded the following formula: autoanalyzer Na (mmol/L) = 20.7 + (0.9 × ABGA Na [mmol/L]) (Fig. 2).

**Table 1:** Demographic characteristics and laboratory data of the study infants

Patient characteristics (*N* = 1041)	Average	(Range)
Gestational age (weeks)	31.4 ± 3.5	(23–40)
Postmenstrual age (weeks)	33.2 ± 3.2	(22.6–44.3)
Time of sample collection (days)	10.0 ± 12.9	(1–56)
Body weight (g)	1605.4 ± 513.3	(450–2490)
Auto-analyzer sodium (mmol/L)	138.8 ± 4.7	(119.0–158.0)
ABGA sodium (mmol/L)	134.6 ± 3.5	(117.5–152.2)
Serum protein (g/dL)	4.9 ± 0.6	(2.2–6.8)
Serum albumin (g/dL)	3.3 ± 0.4	(1.4–4.4)

Data are presented as mean ± SD

*ABGA* arterial blood gas analyzer

Among the patients with normal sodium ion levels and hypernatremia according to the indirect method, the mean differences in the sodium ion levels between the two methods were 4.2 ± 2.9 and 9.9 ± 3.7 mmol/L, respectively (*P* < 0.001), exceeding the acceptable limit defined in the US CLIA 1988 rules. Although the mean difference of 1.0 mmol/L in patients with hyponatremia was within the acceptable limit defined in the US CLIA 1988 rules, it was statistically significant (*P* < 0.001) (Table 2). The incidence of hyponatremia identified using the ABGA was 51.9 % (541/1041), while the incidence of hyponatremia identified using the autoanalyzer was only 14.0 % (146/1041).

**Table 2:** Classified analysis of the differences in the sodium ion levels between the arterial blood gas analyzer (ABGA) and the autoanalyzer

Indirect Method (mmol/L)	Sodium concentration (Auto-analyzer – ABGA)		
	Cases (*N* = 1041)	Mean	*P*- value
<135	146	1.0 ± 2.7	<0.001
135–145	815	4.2 ± 2.9	<0.001
>145	80	9.9 ± 3.7	<0.001
Total	1041	4.2 ± 3.6	<0.001

Data are presented as mean ± SD

In the univariate analysis, a significant association was found between a sodium ion difference of >4 mmol/L and the plasma protein level. In the multiple logistic regression analysis, the plasma protein level was identified as an independent risk factor for a sodium ion level difference of >4 mmol/L between the methods (odds ratio = 2.870, *P* < 0.001) (Table 3).

**Table 3:** Unadjusted and adjusted odds ratios for the risk of exceeding the upper limit of the sodium ion level (4 mmol/L) according to the protein and albumin levels [15–17]

Variable	Cases (*N* = 1041)	Unadjusted			Adjusted		
		OR	95 % CI	*P* value	OR	95 % CI	*P* value
Gestational age (wk)							
< 34	700	0.914	0.705–1.185	0.498	0.821	0.607–1.112	0.203
≥ 34	341	1.000	(Reference)		1.000	(Reference)	
Birth weight (g)							
< 1500	442	1.071	0.837–1.370	0.585	1.112	0.823–1.503	0.490
≥ 1500	599	1.000	(Reference)		1.000	(Reference)	
Protein (g/dL)							
< 4.3	147	2.565	1.779–3.698	<0.001	2.870	1.920–4.290	<0.001
≥ 4.3	894	1.000	(Reference)		1.000	(Reference)	
Albumin (g/dL)							
< 2.2	1024	0.981	0.435–2.210	0.963	0.472	0.197–1.130	0.920
≥ 2.2	17	1.000	(Reference)		1.000	(Reference)	
Sampling day (d)							
≤ 7	708	1.360	1.045–1.769	0.022	1.326	0.990–1.775	0.058
> 7	333	1.000	(Reference)		1.000	(Reference)	

*OR* odds ratio, *95* % *CI* 95 % confidence interval

## Discussion

In the present study, we found that the sodium ion levels in preterm infants simultaneously determined using an ABGA and an autoanalyzer were significantly different.

In correlation analysis, a close relationship was identified in the sodium ion levels between the ABGA and the autoanalyzer (*r*^2^ = 0.44). However, the mean difference (4.2 mmol/L) exceeded the limit imposed by the US CLIA 1988 rules (4 mmol/L) [4]. Additionally, the mean difference was highest in patients with hypernatremia (9.9 mmol/L). In multiple logistic regression analysis, the plasma protein level was identified as an independent risk factor for a sodium ion level difference of >4 mmol/L between the methods.

The difference in the sodium ion levels between the ABGA and the autoanalyzer might be explained by various factors. Collection and transportation of the samples might have contributed to the difference in the sodium ion levels. However, the heparin syringe used in this study for sample collection for the ABGA includes lyophilized lithium heparin (spray-dried calcium-balanced lithium heparin), and this syringe has been shown to rarely cause bias [5, 9]. The blood collection tube used in this study for sample collection for the autoanalyzer is a microtube containing the clot activator SST^TM^ Gel. Because the microtube is filled with only 400–600 μL of blood, bias is minimal.

Previous studies have shown that solid elements of blood, including proteins and lipids, can cause a false diagnosis of hyponatremia (pseudohyponatremia) via an electrolyte exclusion effect when an indirect method is used [4, 10, 11]. It is known that younger and smaller infants tend to have low protein levels initially. In the present study, the sodium ion levels determined using the indirect method were higher than those determined using the direct method. Moreover, the difference in the sodium ion levels exceeded 4 mmol/L more often in infants with low plasma protein level (<4.3 g/dL) than in those with high plasma protein level (≥4.3 g.dL). Previous studies in adults showed that the difference in the sodium ion levels between the two methods can be large in patients with hypoalbuminemia and low serum total protein [6, 12].

The sodium ion levels determined using the ABGA and the autoanalyzer were not equivalent in premature infants admitted to the NICU. Therefore, clinicians should be careful when diagnosing sodium ion imbalance and devising treatment. In particular, the difference between the two methods can lead to inaccurate anion gap measurement, resulting in incorrect determination of metabolic status [13]. For correcting inconsistencies for adults with abnormal protein levels, especially for those with multiple myeloma, some hospitals use a compensation equation [14]. However, there was no previous study on the difference in the sodium ion levels between the direct and indirect methods in premature infants, and the critical difference in the neonatal period has not been determined. Our study clarifies the difference in the sodium ion levels between the direct and indirect methods in premature infants. The use of a compensation equation in each hospital is a possible solution to overcome the inconsistency in sodium ion levels assessed using the direct and indirect methods. Additionally, the direct method might be preferable [4, 6]. The direct method is not influenced by the solid components of blood, which could lead to a false result, and does not require dilution of the sample; therefore, the direct method is considered to be more accurate and consistent for the evaluation of sodium ion levels than the indirect method [12].

The main limitation of the present study is its retrospective design. However, the samples analyzed using the ABGA and the autoanalyzer were obtained at the same time points. Another limitation is that the levels of lipid, one of the solid components of blood, were not measured routinely, precluding the analysis of the effect of lipid on the difference in sodium levels determined with the two methods. Despite these limitations, the results can be used for more precise determination of sodium levels in preterm infants, and are therefore important.

## Conclusion

The sodium ion levels determined using the ABGA and the autoanalyzer might not be equivalent in premature infants admitted to the NICU. Although the direct method has been shown to be suitable for assessing sodium ion levels in previous studies, these studies were performed in adults; therefore, clinicians should be careful when diagnosing sodium ion imbalance in premature infants and providing treatment. Further studies are needed to determine the factors responsible for the inconsistency in the sodium ion levels between the direct and indirect methods.

## Abbreviations

ABGA: arterial blood gas analyzer; NICU: neonatal intensive care unit; US CLIA: The United States clinical laboratory improvement amendments

## Additional file

Additional file 1: The datasets supporting the conclusion of this study. (XLSX 820 kb)
